# Identification and bioinformatics analysis of lncRNAs in serum of patients with ankylosing spondylitis

**DOI:** 10.1186/s12891-024-07396-z

**Published:** 2024-04-15

**Authors:** Jianqiang Kou, Yongchen Bie, Mingquan Liu, Liqin Wang, Xiangyun Liu, Yuanliang Sun, Xiujun Zheng

**Affiliations:** 1 Department of Spinal Surgery, The Affiliated Hospital of Qingdao University, Qingdao, 266000 Shandong China; 2 Department of Operating Room, The Affiliated Hospital of Qingdao University, Qingdao, 266000 Shandong China; 3 Department of Rheumatology, The Affiliated Hospital of Qingdao University, Qingdao, 266000 Shandong China

**Keywords:** Differentially expressed lncRNA, RNA sequencing, Serum, Ankylosing spondylitis

## Abstract

**Objectives:**

The aim of this study was to explore the long non-coding RNA (lncRNA) expression profiles in serum of patients with ankylosing spondylitis (AS). The role of these lncRNAs in this complex autoimmune situation needs to be evaluated.

**Methods:**

We used high-throughput whole-transcriptome sequencing to generate sequencing data from three patients with AS and three normal controls (NC). Then, we performed bioinformatics analyses to identify the functional and biological processes associated with differentially expressed lncRNAs (DElncRNAs). We confirmed the validity of our RNA-seq data by assessing the expression of eight lncRNAs via quantitative reverse transcription polymerase chain reaction (qRT-PCR) in 20 AS and 20 NC samples. We measured the correlation between the expression levels of lncRNAs and patient clinical index values using the Spearman correlation test.

**Results:**

We identified 72 significantly upregulated and 73 significantly downregulated lncRNAs in AS patients compared to NC. qRT-PCR was performed to validate the expression of selected DElncRNAs; the results demonstrated that the expression levels of MALAT1:24, NBR2:9, lnc-DLK1-35:13, lnc-LARP1-1:1, lnc-AIPL1-1:7, and lnc-SLC12A7-1:16 were consistent with the sequencing analysis results. Enrichment analysis showed that DElncRNAs mainly participated in the immune and inflammatory responses pathways, such as regulation of protein ubiquitination, major histocompatibility complex class I-mediated antigen processing and presentation, MAPkinase activation, and interleukin-17 signaling pathways. In addition, a competing endogenous RNA network was constructed to determine the interaction among the lncRNAs, microRNAs, and mRNAs based on the confirmed lncRNAs (MALAT1:24 and NBR2:9). We further found the expression of MALAT1:24 and NBR2:9 to be positively correlated with disease severity.

**Conclusion:**

Taken together, our study presents a comprehensive overview of lncRNAs in the serum of AS patients, thereby contributing novel perspectives on the underlying pathogenic mechanisms of this condition. In addition, our study predicted MALAT1 has the potential to be deeply involved in the pathogenesis of AS.

**Supplementary Information:**

The online version contains supplementary material available at 10.1186/s12891-024-07396-z.

## Introduction

Ankylosing spondylitis (AS) is a common chronic inflammatory disease with an autoimmune etiology [1]. AS often affects the axial joints, including the sacroiliac joint and spine, and induces new bone formation and ultimately ankylosis [2, 3]. In most cases, the initial stage of AS is relatively insidious, which makes an early diagnosis difficult and leads to delayed treatment [4]. Although HLA-B27 misfolding [5], certain cytokines [6], and autophagy [7] are associated with AS progression, the etiology and pathogenesis of AS remain largely unclear. A better understanding of the underlying factors will help us to better understand the origins and progression of AS, and will help us identify new biomarkers or therapeutic targets.

Long non-coding RNAs (lncRNAs) are transcripts longer than 200 nucleotides that lack the protein-coding capacity, but can post-transcriptionally regulate the expression of certain genes [8, 9]. In recent years, some important lncRNAs have been observed in AS, which may be involved in AS pathogenesis. For example, MEG3 was found to sponge miR-146a and aberrant MEG3 downregulation could promote the inflammatory response through increasing the abundance of miR-146a [10]. Another study revealed that downregulation of LOC645166 promotes the NF-κB signaling via reduced blocking recruitment of the IKK complex to K63-linked polyubiquitin chains [11]. H19 is significantly upregulated in AS and mediates the inflammatory processes by acting as a competing endogenous RNA (ceRNA) in the miR22-5P-VDR-IL-17A/IL-23 axis in peripheral blood mononuclear cells [12]. Although several lncRNAs play important roles in diverse biological processes, the function and biological relevance of the vast majority of lncRNAs remain unclear. To the best of our knowledge, the expression profiles of lncRNAs, along with their potential biological functions, in the peripheral serum of patients diagnosed with AS still unclear in the current academic literature.

In the present study, we aimed to identify novel DElncRNAs for research and therapeutic purposes, and explored the signaling pathways underlying the DElncRNAs. To this end, we performed high-throughput RNA sequencing and bioinformatics analyses to identify the lncRNA expression profiles. Furthermore, the relevant pathways and biological functions of these RNAs were investigated using Gene Ontology (GO), Kyoto Encyclopedia of Genes and Genomes (KEGG), and Reactome analyses. Finally, a regulatory network was constructed based on the identified lncRNAs.

## Materials and methods

### Patients and samples

We enrolled 23 AS patients who presented to the Department of Rheumatology and Immunology of the Affiliated Hospital of Qingdao University (Qingdao, China) between December 2020 and December 2021. Our study included participants aged at least 18 years who were diagnosed according to the New York criteria established in 1984 [13]. The participants were initial diagnosis and excluded if they had cancer, diabetes, cardiovascular diseases, inflammatory disease, or other autoimmune diseases. All of the patients had not received treatment with non-steroidal anti-inflammatory drugs or biologics. Similarly, we recruited 23 normal controls (NC) who underwent routine physical examination at the hospital. Almost 5 mL of peripheral blood was obtained from each participant by vein puncture and collected in a plain tube. The serum was immediately separated by centrifugation (3000 g; 10 min) at 4˚C. Then, the supernatant was stored in RNase-free centrifuge tubes at − 80◦C until use. RNA-sequencing was performed on serum samples from three AS patients and three NC. Additional 20 pairs of serum samples (from AS and NC groups) were analyzed by quantitative reverse transcription polymerase chain reaction (qRT-PCR). Our study was approved by the Affiliated Hospital of Qingdao University Research Ethics Committee (approval number: QYFY WZLL 27251). Written informed consent was obtained from all participants.

### RNA extraction and high-throughput sequencing

Extractions of total RNA from three pairs of AS and normal serum were performed by the TRIzol reagent (Invitrogen; Termo Fisher Scientifc, Inc., Waltham, MA, USA) according to the kit instructions. Subsequently, the concentration and integrity of the isolated RNA were assessed using a Qubit 3.0 Fluorometer and an Agilent 2100 Bioanalyzer before library construction and sequencing. An RNA library was constructed using the SMARTer Stranded Total RNA-Seq kit v.2 (Takara Bio USA, Mountain View, CA, USA) [14]. Briefly, after the depletion of ribosomal RNA, the fragmented RNA was reversely transcribed into cDNA. Afterward, A-tailing and adapter ligation were performed with purified cDNA. After that, the purified, adapter-ligated DNA was amplified. The cDNA fragments were further purified and enriched with PCR to create the final cDNA libraries, which were analyzed by the Agilent 2100 Bioanalyzer to confirm the quality and concentration. Sequencing was performed in a 150-bp paired-end run (PE150) using the NovaSeq 6000 system (Illumina, San Diego, CA, USA).

### RNA-seq data analysis and differential expression analysis

The reads were mapped to the latest UCSC transcript set using Bowtie2 [15], and the lncRNA expression level was estimated using RSEM [16] and Lncipedia (http://www.lncipedia.org). TMM (trimmed mean of M-values) was used to standardize the gene expression. Differentially expressed genes were identified using the edgeR software [17]. LncRNAs with greater than 1.5-fold change in expression levels and *p* < 0.05 were considered differentially expressed.

### Gene Ontology (GO), Kyoto Encyclopedia of Genes and Genomes (KEGG), and Reactome analyses

To explore the potential functional roles of DElncRNAs, we conducted GO, KEGG, and Reactome enrichment analyses of the cis-regulated genes using the R package clusterProfiler [18]. GO analysis was used to investigate the biological functions based on DElncRNAs. This analysis classifies functions according to the three following aspects: biological process, cellular component and molecular function. A Fisher's exact test was performed to classify the GO category. The lower the *p*-value signified more the GO term (*p* value < 0.05). KEGG [19] and Reactome enrichment analyses were used to predict the pathways related to the cis-regulated genes of DElncRNAs. For further analyses we selected the top ten most significant pathways for each analyzed DElncRNA. *P*-values < 0.05 were considered to indicate statistical significance in the KEGG and Reactome analyses.

### qRT-PCR validation assay

To confirm the reliability of the RNA sequencing data, we selected the top four upregulated and four downregulated DElncRNAs for qRT-PCR validation. Total RNA was extracted from serum using TRIzol reagent following the manufacturer's instructions. The cDNA was synthesized using the Prime Script RT reagent kit with genomic DNA eraser (TaKaRa, Tokyo, Japan), and qRT-PCR was performed on a LightCycler 480 using SYBR Green Master Mix (TaKaRa, Tokyo, Japan). The relative expression levels of candidate lncRNAs were confirmed using the 2^−ΔΔCt^ method [20]. Glyceraldehyde 3-phosphate dehydrogenase (GAPDH) was used as the internal amplification control. The qPCR primer sequences are listed in Table 1.

**Table 1:** Primers used in the present study

Gene	Forward Primer	Reverse Primer
MALAT1:24	TTCCTGTGGCAGGAGAGACAA	TCCTTCGGATGCTTCACTCCA
NBR2:9	AAGAAAAAGGTCAGCTCCACCGA	GGAGGTTTAGCTGACAATTGCG
lnc-MRPS30-7:1	TAGCACCCGTCTCTCCATTTGA	AGCCTGTGTTTGAACCACCAT
lnc-DLK1-35:13	CTCGTCTCCTTCCTGGTTTGG	TCACAGGGCACTTCCATCCTA
lnc-FNTB-4:2	GGGAGTAGCATCCTCCACTCA	GTCCTCTGAGCAAAAGCAGCAA
lnc-LARP1-1:1	TCCACCTATTTTGGCTCCTGTGA	CGTGTTCCTCTATCCCCTGAACT
lnc-AIPL1-1:7	GCCTGCCTACTGGAGCAACTA	ACCTTGGGCACTGATGCTTCT
lnc-SLC12A7-1:16	TTCACACATCCACTCCTTCACA	GAATGAATGGGTGAGATCGTG
GAPDH	GCACCGTCAAGGCTGAGAAC	TGGTGAAGACGCCAGTGGA

### Construction of the lncRNA-miRNA-mRNA competing endogenous RNA (ceRNA) regulatory network

The ceRNA hypothesis reveals a new mechanism of interaction between RNAs, which represents a new regulatory mode of gene expression. We used Cytoscape 3.9.1 to construct a lncRNA-miRNA-mRNA network based on the ceRNA hypothesis and explored their interactions. Briefly, the DElncRNAs were selected to predicted the target miRNA using miRanda v3.3a, and the miRNA-mRNA interaction was predicted using the R package multiMiR [21]. Finally, we constructed the ceRNA network using two clear DElncRNAs and retaining lncRNA-miRNA-mRNA ternary relationships that shared miRNAs.

### Statistical analysis

Statistical analysis was performed using SPSS software (version 26.0). Normally distributed data are expressed as the mean ± standard deviation, and nonnormally distributed data are presented as the median (interquartile range). The differences between two groups were assessed using independent-sample t-tests or the Wilcoxon rank-sum test. The chi-square test or Fisher’s test was used for categorical variables. Spearman correlation coefficient was analyzed to calculate the correlation between the serum levels of DElncRNAs and other variables.* P* < 0.05 was considered statistically significant.

## Results

### Differentially expressed profiles of the serum lncRNA by RNA sequencing

**Fig. 1:**
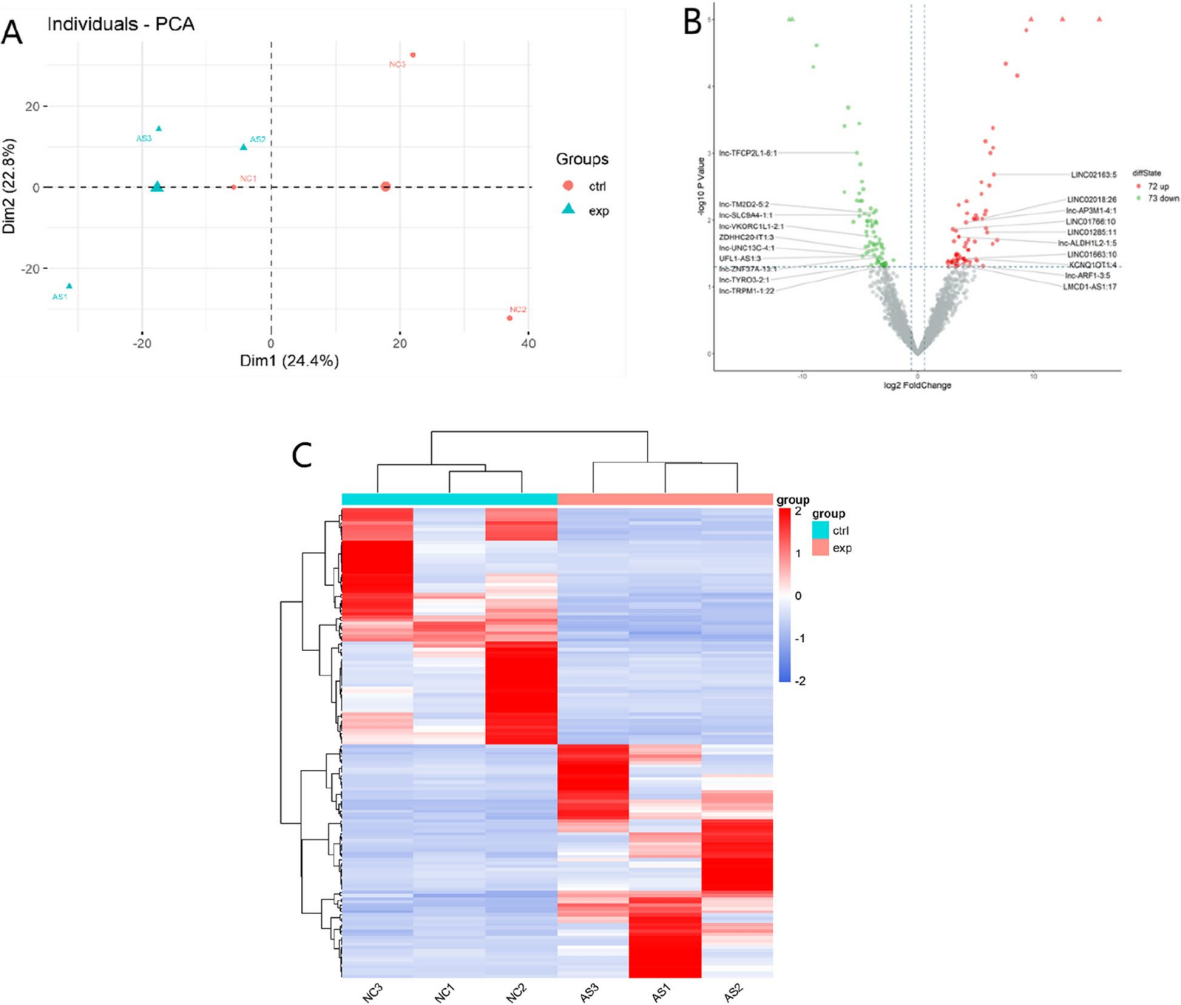
RNA-seq data of differential lncRNA expression profiles in serum from AS patients and NC. **A** PCA plot of lncRNA expression profiles. **B** Volcano plot showing differentially expressed lncRNAs. **C** Heatmap of differentially expressed genes between the AS and NC groups. AS = ankylosing spondylitis, lncRNA = long non-coding RNA, PCA = principal component analysis

The similarities in samples are displayed using principal component analysis (PCA) plot (Fig. 1A). The volcano graph was used to visualize the differences between AS and NC groups (Fig. 1B). We identified 145 lncRNAs (including 72 upregulated and 73 downregulated lncRNAs) that were differentially expressed between the two participant groups. Furthermore, heatmap analyses displayed a similar intra-group clustering and inter-group distinction of DElncRNAs (Fig. 1C). The top 10 up- and downregulated lncRNAs are listed in Table 2.

**Table 2:** Top 10 upregulated and 10 downregulated differentially expressed lncRNAs

Gene name	logFC	*P*.Value	Updown	chrom
MALAT1:24	15.6905991	3.58815E-10	Up	chr11
NBR2:9	12.51702537	6.07368E-07	Up	chr17
lnc-MRPS30-7:1	9.785134841	8.83671E-06	Up	chr5
lnc-DLK1-35:13	9.373592526	1.44424E-05	Up	chr14
lnc-SUMF1-8:1	8.598813714	6.88802E-05	Up	chr3
lnc-CREBRF-1:1	7.613575174	4.57796E-05	Up	chr5
lnc-FBXO42-1:1	6.858975753	0.019924806	Up	chr1
LINC02163:5	6.580962104	0.002085532	Up	chr5
lnc-SAFB-1:1	6.501328558	0.000831325	Up	chr19
lnc-TBL1XR1-6:2	6.483841732	0.000421475	Up	chr3
lnc-TFCP2L1-6:1	-5.276067234	0.000985496	Down	chr2
LINC00649:41	-5.28710823	0.005060239	Down	chr21
lnc-RPS24-3:24	-5.593525554	0.010571416	Down	chr10
lnc-FNTB-4:1	-6.01315169	0.000207927	Down	chr14
lnc-DTWD2-14:3	-6.338357236	0.000394406	Down	chr5
lnc-COMMD10-10:2	-6.354331685	0.003832003	Down	chr5
lnc-FNTB-4:2	-8.757845971	2.44365E-05	Down	chr14
lnc-LARP1-1:1	-9.041578948	5.14405E-05	Down	chr5
lnc-AIPL1-1:7	-10.86646645	5.11705E-06	Down	chr17
lnc-SLC12A7-1:16	-11.13759228	3.6677E-06	Down	chr5

### Pathway enrichment analyses of DElncRNAs

**Fig. 2:**
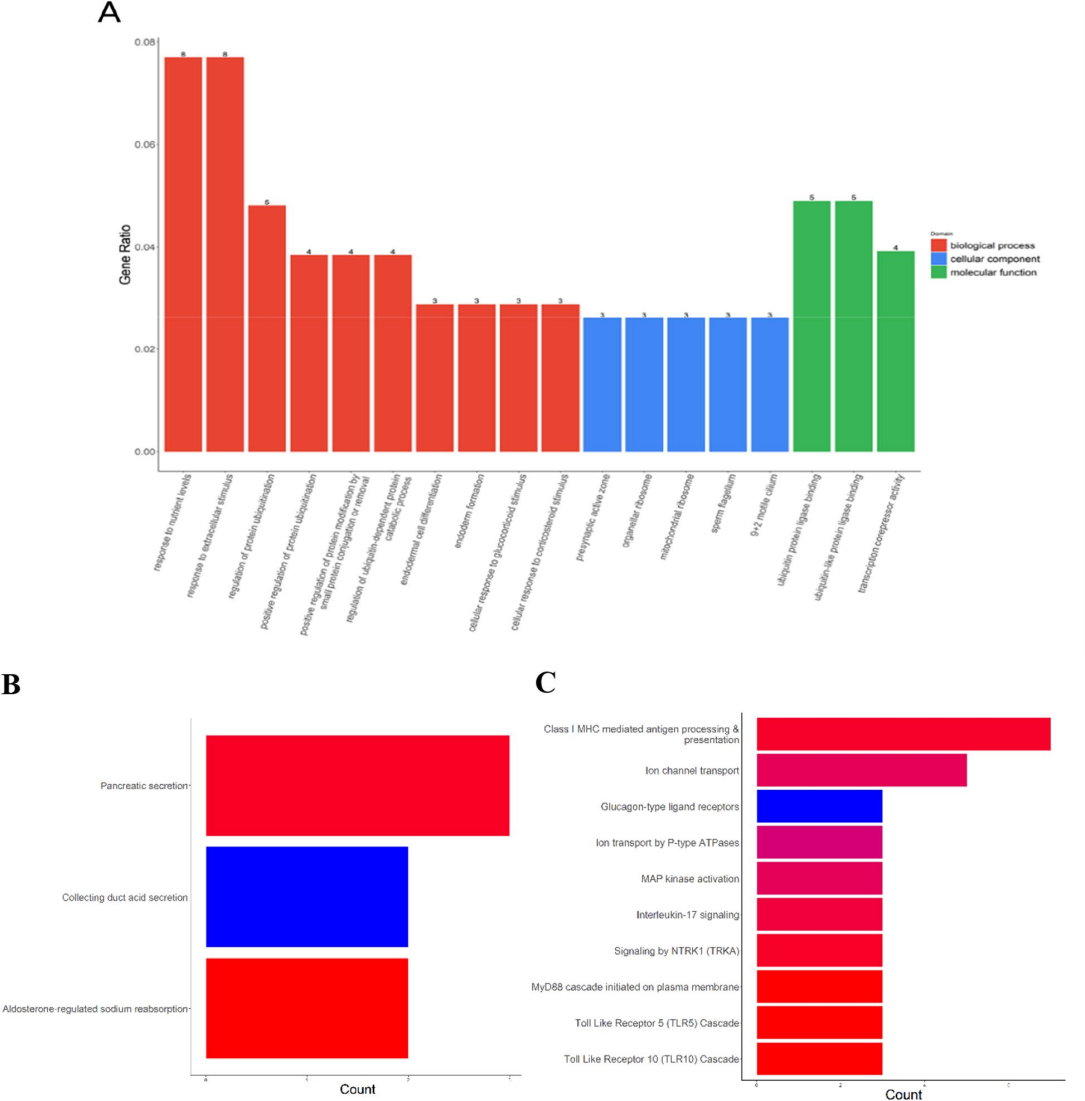
Functional enrichment analysis of differentially expressed lncRNAs. **A** GO enrichment analysis, **B** KEGG enrichment analysis, and (**C**) Reactome analysis of the differentially expressed lncRNAs. lncRNA, long non-coding RNA; GO, Gene Ontology; KEGG, Kyoto Encyclopedia of Genes and Genomes

The potential functional roles of these lncRNAs were assessed via GO, KEGG, and Reactome enrichment analyses. GO analysis was used to identify key biological processes (BPs), cellular components (CCs), and molecular functions (MFs) enriched by these lncRNAs (Fig. 2a). Significantly enriched BPs included the response to nutrient levels, response to extracellular stimulus, regulation of protein ubiquitination, and regulation of ubiquitin-dependent protein catabolic process, while significantly enriched CCs terms included presynaptic active zone, and significantly enriched MFs included ubiquitin protein ligase binding.

In the KEGG analysis, three pathways were found to be significantly enriched for these lncRNAs, including pancreatic secretion, collecting duct acid secretion, and aldosterone-regulated sodium reabsorption (Fig. 2b). To validate the pathways related to DElncRNAs, we identified the top 10 pathways based on Reactome analysis (Fig. 2c). The Reactome pathway analysis revealed that the DElncRNAs were highly associated with immune and inflammation-related pathways in AS patients, including major histocompatibility complex (MHC) class I-mediated antigen processing and presentation, MAP kinase activation, and interleukin-17 signaling pathways.

### qRT-PCR validation of lncRNA expression

**Fig. 3:**
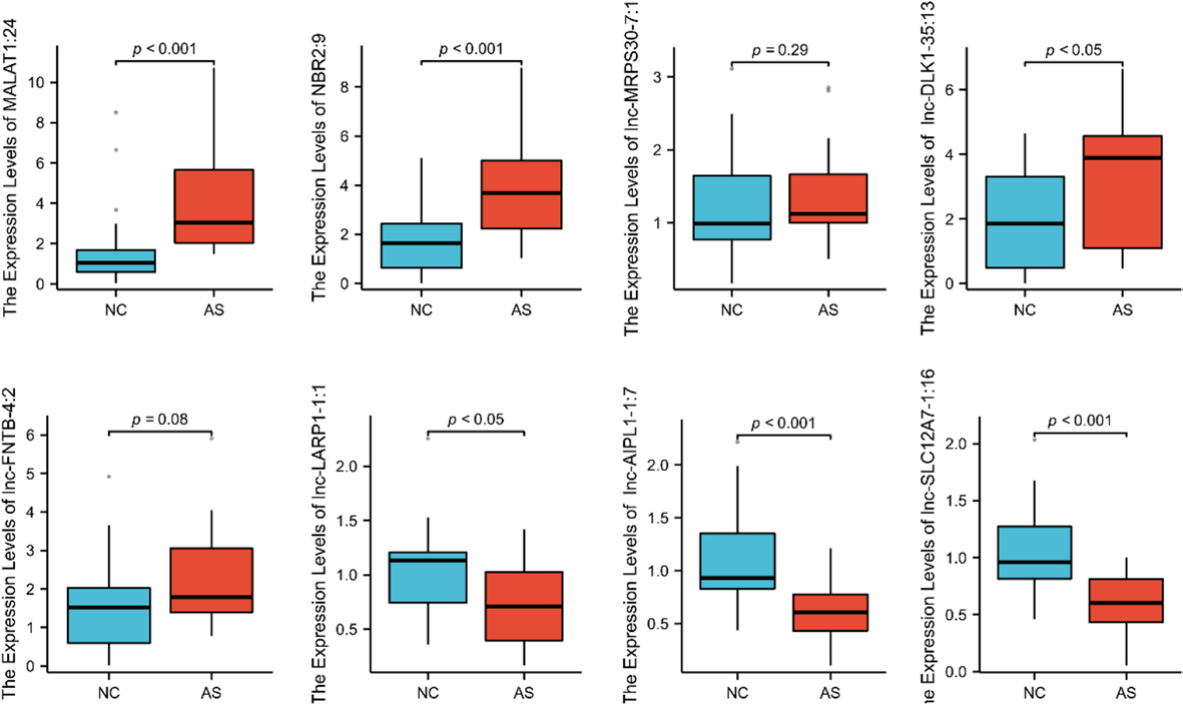
qRT-PCR-based validation of the expression of eight differentially expressed lncRNAs in the serum of NC and AS patients

To confirm the results obtained by RNA-Seq analysis, we selectively performed RT-qPCR analysis of four most significantly upregulated and four most significantly downregulated lncRNAs (MALAT1:24, NBR2:9, lnc-MRPS30-7:1, lnc-DLK1-35:13, lnc-FNTB-4:2, lnc-LARP1-1:1, lnc-AIPL1-1:7, and lnc-SLC12A7-1:16) in 20 AS patients and 20 NC. The relative lncRNA expression levels of MALAT1:24, NBR2:9, and lnc-DLK1-35:13 were significantly higher in serum samples from AS patients than those from NC. The expression levels of lnc-LARP1-1:1, lnc-AIPL1-1:7, and lnc-SLC12A7-1:16 were significantly lower in AS patients than those in NC. However, the levels of lnc-MRPS30-7:1 and lnc-FNTB-4:2 were not significantly different between AS patients and NC (Fig. 3), which is inconsistent with the RNA-seq data.

### Construction of the lncRNA-miRNA-mRNA ceRNA regulatory network

**Fig. 4:**
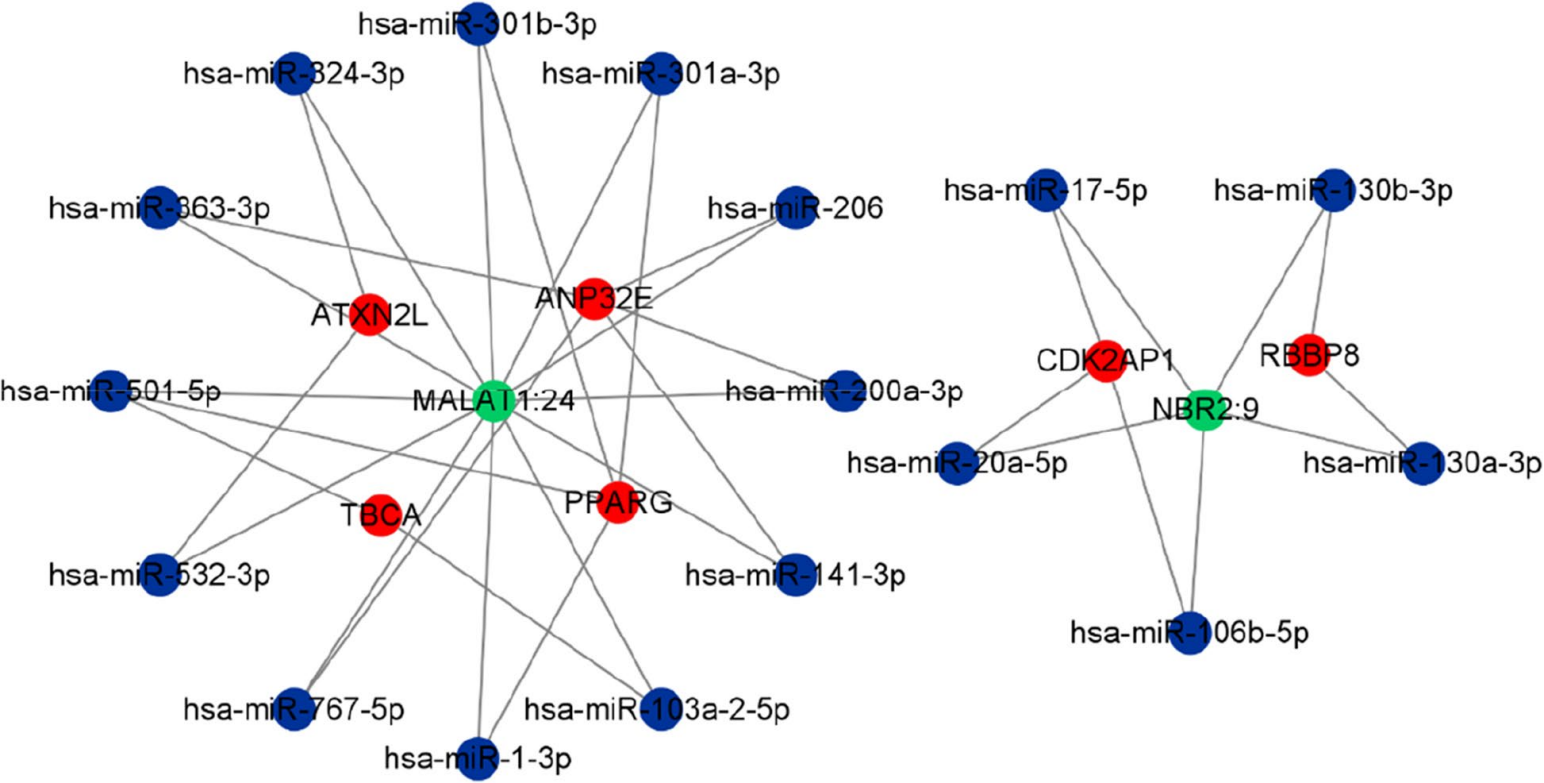
Construction of the dysregulated lncRNA‐miRNA‐mRNA network in AS patients. Green, blue, and red nodes represent the lncRNAs, miRNAs, and mRNAs, respectively

To further interrogate the functional role of the differentially expressed serum lncRNAs as competing with miRNA to repress its regulation of the target mRNA, a ceRNA network was constructed. As shown in Fig. 4, this network contained two validated DE lncRNAs, 17 miRNAs that may bind to these lncRNAs, and six downstream target mRNAs of the miRNAs. The results demonstrated that several critical lncRNAs can be used as ceRNAs to regulate gene expression through sponge miRNAs and play a key role in the pathological processes underlying AS.

### Correlation between serum lncRNA expression and clinical characteristics of AS

**Fig. 5:**
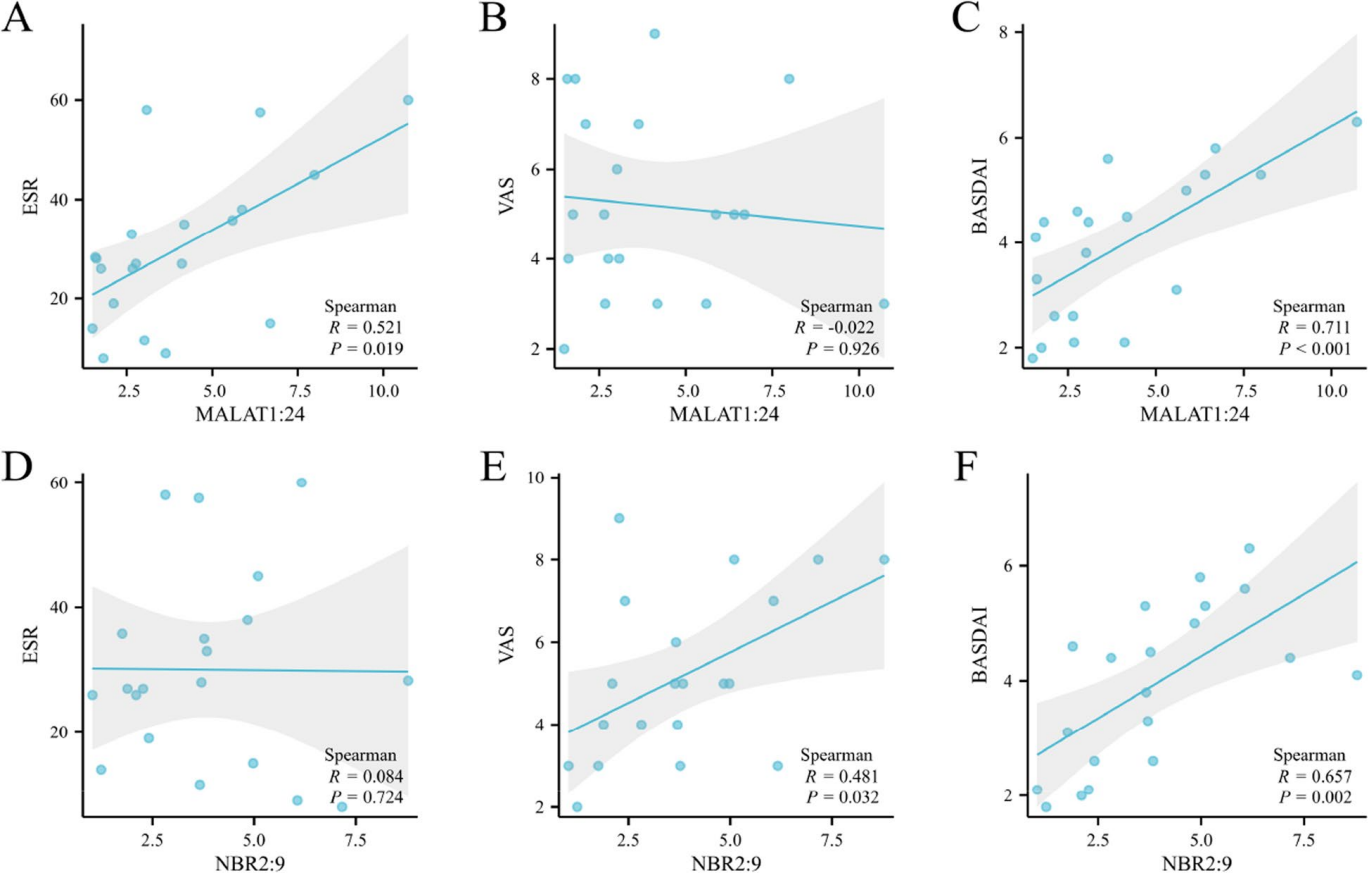
Correlations between lncRNA expression profiles and AS clinical disease activity

We performed Spearman’s analysis to further explore the correlation between serum MALAT1:24, NBR2:9 and the clinical characteristics of AS (Fig. 5). In this study, the expression level of serum MALAT1:24 was positively correlated with ESR (*r* = 0.521, *P* = 0.019) and BASDAI staging (*r* = 0.711, *P* < 0.001), the expression level of serum NBR2:9 was positively correlated with BASDAI (*r* = 0.657, *P* = 0.002) and VAS (*r* = 0.481, *P* = 0.032). These results indicated that MALAT1:24 and NBR2:9 may be related to the AS initiation and progression.

## Discussion

Ankylosing spondylitis (AS) is a multifaceted ailment encompassing numerous factors [22]. Various treatment approaches have been devised to address AS; nevertheless, the majority of therapeutic interventions have proven inadequate in attaining desirable results. Furthermore, AS seriously affects the physical and mental health and quality of life of patients. Consequently, the timely identification and prompt initiation of treatment are imperative. The importance of lncRNAs in various processes, such as transcriptional, post-transcriptional, and epigenetic regulation, is well known [23]. Some lncRNAs are key determinants of metabolic activity, development, evolution, and disease pathophysiology [24]. Moreover, a growing body of evidence suggests that the dysregulation of lncRNAs plays an important role in the occurrence and progression of multiple immune-mediated inflammatory diseases, including AS [25]. However, only a small proportion of lncRNAs implicated in the AS pathogenesis has been identified, and no studies have investigated the lncRNAs present in the serum of AS patients. Hence, we conducted the present study to investigate the lncRNA expression profiles in the serum of AS patients, which may help to understand the pathophysiology and develop early diagnostic techniques and treatments for AS.

In our study, PCA plot and heatmap plot analyses illustrated that lncRNA expression profiles could distinguish AS patients from NC. Furthermore, the volcano plot showed that there were 145 DElncRNAs in AS patients compared to NC; among them, 72 lncRNAs were downregulated and 73 were upregulated. To explore the functional roles of the differentially expressed genes, we conducted GO, KEGG, and Reactome pathway analyses based on cis-regulation genes of DElncRNAs. The results indicate that DElncRNAs primarily participate in the regulation of protein ubiquitination, MHC class I-mediated antigen processing and presentation, MAP kinase activation, and interleukin-17 (Th17) signaling pathways, suggesting that DElncRNAs were enriched in pathways related to immune and inflammatory responses. The physiological function of the MHC class I molecules is to present antigenic or mutated peptides to the antigen receptors of CD8 + T cells and engage the receptors of natural killer cells for broad immunosurveillance against many pathogenic conditions [26, 27]. In line with this, a number of studies have reported a strong association between MHC class I allele human leukocyte antigen B27 and AS [5]. Based on previous evidence, the MAPK signaling pathway was implicated in the development of Th1 cells and macrophage activation [28]. Lin et al. found miR-134-5p inhibits osteoclastogenesis through the miR-134-5p/Itgb1/MAPK pathway [29]. Moreover, Pedersen et al. demonstrated that IL-17 is involved in the pathological mechanism of AS [30], especially in the occurrence and development of enthesitis, bone erosions, and bone formation [31, 32]. Some of the identified pathways, including the regulation of protein ubiquitination and signaling by NTRK1 (TRKA), have not been studied previously in the context of AS and thus warrant further investigation.

We validated the differential expression of four up- and downregulated highest fold-change lncRNAs via qRT-PCR to confirm the reliability of our RNA-seq data. This approach confirmed that six of the lncRNAs were differentially expressed in the expected directions (all *p* < 0.05). However, the levels of lnc-MRPS30-7:1 (*p* = 0.29) and lnc-FNTB-4:2 (*p* = 0.08) were not in line with the sequencing results. To determine the potential downstream targets of lncRNAs, we used the two most significantly upregulated lncRNAs (MALAT1 and NBR2) to construct a ceRNA. We found that MALAT1 may regulate the AS pathogenesis through the MALAT1/miR-1-3p/PPARG axis. Previous studies have shown that miR-1-3p is closely related to the pathogenesis of autoimmune diseases as well as several other diseases. As an example, the expression levels of MALAT1 and miR-1-3p were significantly higher in newly diagnosed patients with rheumatoid arthritis than NC [33]. Some studies have also shown that overexpression of miR-1-3p might facilitate Th17 differentiation by inhibiting the expression of ETS1 in naïve CD4^+^ T cells [34]. Peroxisome proliferator activated receptor gamma (PPARG) is a part of the subfamily of peroxisome proliferators activated receptors, which also includes PPARα and PPARβ/δ. This subfamily of receptors, especially PPARG, inhibits the key pro-inflammatory genes, such as NF-kB and TNFα, and the interleukins IL-1a and IL-6 [35]. In a study by Lin et al., lipocalin 2, which was regulated by PPARG, was found to be a potential pathway involved in concurrent ankylosis and inflammation in AS and inflammatory bowel diseases [36]. An additional study found that PPARG was highly expressed in peripheral serum of AS patients, which was validated by qRT-PCR [37]. Therefore, we speculate that there is a potential regulatory relationship between PPARG and MALAT1 in the serum of patients with AS.

MALAT1 is a large (6.5 kb), nuclear-enriched, and highly conserved lncRNA, which has important regulatory functions related to inflammation and is associated with autoimmune diseases. Increasing evidence indicates that MALAT1 acts as a competing endogenous RNA (ceRNA) to reduce the repression effect of miRNA on the expression of certain inflammatory factors [38]. Zhou et al. suggested that the downregulation of lncRNA MALAT1 hindered the inflammation of microglial cells and prevented the progression of acute spinal cord injury via the modulation of the miR-199b/IKKβ/NF-κB signaling pathway [39]. Pathological bone formation is another pivotal mechanism involved in the pathology of AS. In vitro knockdown of MALAT1 suppresses the proliferation of the human osteoblast cell line hFOB 1.19 [40], and MALAT1 sponging of the microRNA miR-30 promotes the osteoblastic differentiation of mesenchymal stem cells by inducing RUNX2 expression [41]. Furthermore, the downregulation of lncRNA MALAT1 decreased miR-558-mediated GSDMD expression to enhance cell viability and suppress apoptosis and pyroptosis of chondrocytes in AS [42]. Therefore, MALAT1 plays a unique role in the AS pathogenesis, although additional research is needed to understand the mechanisms whereby the candidate lncRNAs impact disease progression.

Our results provide new insights into the underlying mechanisms of AS. However, some limitations of our study should be considered. First, the sample size was relatively small, and all cases were recruited from a single center. Therefore, large-scale, multi-center cohort studies are needed to confirm the accuracy of our RNA-seq results. Second, follow-up studies using in vitro experiments and animal models are required to determine the underlying regulatory mechanisms of the DElncRNAs in AS pathogenesis.

### Supplementary Information

**Additional file 1.** Patient characteristics.

## Data Availability

The datasets generated and/or analysed during the current study are available in the Gene Expression Omnibus (GEO) repository, under accession number GSE205812 that are publicly accessible at http://www.ncbi.nlm.nih.gov/geo.
