# Supplementary material for: Identification and bioinformatics analysis of lncRNAs in serum of patients with ankylosing spondylitis
Source: BMC Musculoskelet Disord. 2024 Apr 15;25:291. doi: 10.1186/s12891-024-07396-z (PMC11017588; doi:10.1186/s12891-024-07396-z)
Supplement: Supplementary file 1 — Additional file 1. Patient characteristics. [file 12891_2024_7396_MOESM1_ESM.docx]

**Supplementary information**

Patient characteristics

Table 1 Study population clinical characteristics

| Indexes | AS | NC | *P* value |
| --- | --- | --- | --- |
| Sex (M/F) | 20/3 | 20/3 | 1.000 |
| Age (years) | 31.21 ±7.83 | 32.00 ±7.14 | 0.788 |
| HLA-B27 positive, No. (%) |  |  |  |
| No | 2 (8.7) | NA | NA |
| Yes | 21 (91.3) | NA | NA |
| ESR (mm/h) | 29.40 ±15.06 | NA | NA |
| CRP (mg/dL) | 19.32 ± 10.05 | NA | NA |
| BAS DAI (score) | 4.03 ± 1.39 | NA | NA |
| BASFI (score) | 3.77 ± 1.24 | NA | NA |
| VAS (score) | 5.30 ± 1.94 | NA | NA |

The AS and NC groups did not differ significantly in age or sex
